# Harnessing Sequence Embedding and Ensemble Learning to Identify Antifungal Peptides with Low Hemolytic Risk

**DOI:** 10.1021/acsomega.6c00049

**Published:** 2026-04-27

**Authors:** Chung-Yen Lin, Wen-Chih Cheng, U-Lin Chen, Tzu-Tang Lin, Li-Hang Hsu, Yang-Hsin Shih, I-Hsuan Lu, Ying-Lien Chen, Shu-Hwa Chen

**Affiliations:** † 71554 Institute of Information Science, Academia Sinica, Taipei 115, Taiwan; ‡ Institute of Fisheries Science, College of Life Science, 529873 National Taiwan University, Taipei 10617, Taiwan; § Genome and Systems Biology Degree Program, National Taiwan University, Taipei 10617, Taiwan; ∥ Data Science Program, National Taiwan University, Taipei 10617, Taiwan; ⊥ College of Pharmacy, 15505 University of Florida, Gainesville, Florida 32610, United States; # Department of Plant Pathology and Microbiology, National Taiwan University, Taipei 10617, Taiwan; ∇ Department of Agricultural Chemistry, National Taiwan University, No. 1, Sec. 4, Roosevelt Rd., Taipei 10617, Taiwan; ○ TMU Research Center of Cancer Translational Medicine, 38032 Taipei Medical University, Taipei 110, Taiwan

## Abstract

The increasing prevalence of fungal infections represents a growing threat to human health, driven in part by the misuse of antibiotics and the rising incidence of resistance to conventional antifungal agents. Antifungal peptides (AFPs) have emerged as promising alternatives due to their diverse mechanisms of action and their relatively low propensity to develop resistance. To facilitate the systematic discovery of AFPs, we developed AI4AFP. This computational framework integrates curated antifungal peptide resources with advanced machine learning approaches to predict antifungal potential directly from peptide sequences. Using a comprehensive data set, we constructed a seven-model ensemble that combines multiple sequence encoding strategies, including ProtBERT-BFD, PC6, and Doc2Vec, with diverse learning algorithms, including random forests, support vector machines, convolutional neural networks, and fine-tuned BERT models. This ensemble demonstrated robust performance on an independent test set, achieving 0.94 in accuracy and 0.89 in Matthews correlation coefficient, outperforming existing AFP prediction methods. Importantly, the predicted AFP score is intended to reflect the general antifungal potential rather than species-specific potency. Experimental validation against representative fungal pathogens, including *Candida albicans*, *Candida glabrata*, and *Cryptococcus neoformans*, revealed that peptides with high predicted AFP scores exhibited context-dependent antifungal activity. Several candidates displayed pronounced inhibitory effects against specific species, despite limited activity against others, highlighting the inherent species dependence of antifungal efficacy and supporting the role of AI4AFP as a prioritization tool rather than a species-specific predictor. To complement antifungal prediction, we further developed a hemolysis classifier that incorporates both peptide sequence and applied concentration as continuous inputs, enabling explicit modeling of the dose-dependent nature of hemolytic toxicity. Experimental determination of the minimum concentration inducing 10% hemolysis (MHC_10_) provided an empirical safety reference, enabling antifungal activity to be interpreted alongside concentration-dependent toxicity. All models and validation results are implemented on a user-friendly web server, AI4AFP (https://axp.iis.sinica.edu.tw/AI4AFP), providing an accessible platform for the discovery and prioritization of antifungal peptides, with consideration of both efficacy and safety.

## Graphical abstract

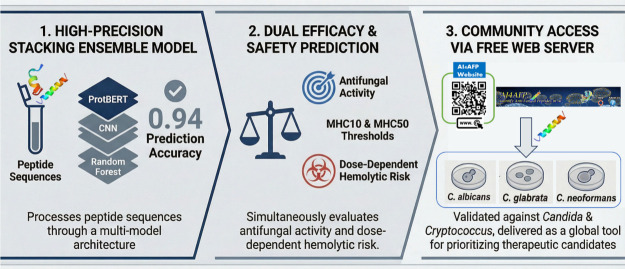

## Introduction

Fungi are ubiquitous microorganisms that are present everywhere and play important roles in the ecosystem, some could be infectious and lethal, threatening human life. Fungal infections affect over a billion people worldwide, and the major fungal species like *Aspergillus*, *Candida*, and *Cryptococcus* cause more than 150 million fatal diseases.^1^ Natural products like penicillins, cephalosporins, and their derivatives have been successfully developed into clinically safe and effective antibiotics for bacterial infections.^2^ In contrast, the discovery of antifungal agents with comparable clinical safety has lagged significantly behind. Problems of antifungal drug resistance have arisen due to misuse and the rapid evolution of pathogens, and the limited diversity of available antifungals further exacerbates these growing concerns. Antifungal peptides (AFPs) are a subset of antimicrobial peptides (AMPs) with properties against pathogenic fungi. Due to the lower host toxicity, broad antimicrobial activity, and modes of action that prevent fungi from developing resistance, AFPs are potential alternatives as new antibiotic agents.^3^ Discovering valuable AFPs from numerous unknown peptides through traditional methods is always time-consuming and expensive. An effective way to accelerate AFP drug development and reduce resource costs is to conduct *in silico* evaluation to predict peptides’ antifungal abilities before further experiments.

In the past decade, AI-based antifungal peptide (AFP) prediction has rapidly evolved in model architecture and feature engineering. Early models, such as SVM-based classifiers,^4^ achieved moderate performance using amino acid composition and physicochemical properties. More recently, deep learning frameworks like Deep-AntiFP,^5^ DeepAF,^6^ and AFP-MFL^7^ have reported test accuracies in the range of 89–94%, integrating evolutionary features, contextual embeddings, and multiview fusion. The iAFPs-EnC-GA^8^ ensemble model and BiTCN-based models^9^ have pushed performance further, achieving up to 94–99% accuracy and AUC values close to 0.98 on independent tests. Innovations such as transform-based evolutionary features, pretrained protein language models, and attention-guided feature fusion have contributed to improved generalization.

Despite these advances, the quality and construction of training data sets remain a critical limitation. Many models rely on peptides annotated as AFPs for positive labels while designating unannotated sequences—including other antimicrobial peptides (AMPs)—as negative examples. This approach risks including peptides with unrecognized antifungal activity in the negative class, introducing labeling bias and compromising model robustness. Developing more rigorous data set-curation strategies that account for such ambiguities will be essential to improving the accuracy and reliability of future AFP prediction models. While recent AI models for antifungal peptide prediction have achieved high performance through deep learning and advanced feature engineering, none have systematically integrated hemolytic safety assessment into the prediction framework.^4,5,8,9^ This underscores a critical gap that a dual-model platform needed to address, directly by jointly evaluating antifungal activity and hemolytic risk.

In recent years, AI-based models have made significant strides in predicting the hemolytic potential of functional peptides. For instance, HAPPENN^10^ employs a neural network classifier with a comprehensive feature set—amino acid composition, dipeptide/tripeptide patterns, physicochemical descriptors, PseAAC, and conjoint triads—achieving an AUC-ROC of 0.90. Other advanced methods leverage sequence-derived features, including traditional encodings,^11^ transformer-based embeddings,^12^ structural descriptors,^13^ and domain-specific embeddings.^14^ Deep learning architectures such as Hybrid Transformer-CNN,^15^ HemoPI2,^16^ and HemoDL^12^ have further elevated prediction performance.

However, a common limitation across these approaches is that they rely solely on peptide-sequence-derived features and ignore concentration as a predictive factor. This overlooks a crucial biological reality—hemolytic activity is concentration-dependent. A safe peptide at low concentrations may become hemolytic at higher doses. This gap remains unaddressed in current AI models, limiting their real-world applicability for therapeutic peptide screening.

To meet the unmet needs mentioned above, we developed AI4AFP, a user-friendly web-based tool that integrates two independent ensemble AI models to assess peptide functionality and safety. The first ensemble model predicts whether a peptide exhibits antifungal activity (AFP), while the second independently evaluates its hemolytic potential, helping users efficiently screen therapeutic peptide candidates.

The AFP activity prediction model was built through three key steps: data curation, sequence encoding, and model training. Positive AFPs were collected from public AMP databases such as CAMP,^17^ DRAMP,^18^ YADAMP,^19^ SATPdb,^20^ and DBAASP^21^ by filtering entries explicitly labeled with “antifungal” activity. Importantly, unlike earlier approaches (e.g., Antifp^4^ and AFPDeep),^22^ we avoided using AMPs lacking antifungal annotation as negative data to prevent potential mislabeling that could bias the model.

For sequence encoding, we applied three complementary methods:

- PC6 encoding,^23^ which encodes six physicochemical properties into a 2D matrix,
- Doc2Vec,^24^ a flexible NLP-based vectorization technique, and
- ProtBert-BFD,^25^ a BERT-derived language model that captures sequence-level semantic features.

These encoded features were used to train several classifiers, including random forest (RF), support vector machine (SVM), and a convolutional neural network (CNN). The best-performing models were combined into an ensemble, yielding high predictive accuracy.

Separately, we developed a novel ensemble model for hemolytic risk prediction, uniquely incorporating both peptide sequence and concentration as input features. This model addresses a major limitation in existing tools, which typically ignore concentration-dependent hemolytic effects. By integrating concentration data, our hemolysis model provides a more biologically relevant and efficient alternative to labor-intensive experimental validation.

Both models were ultimately integrated into the AI4AFP web platform, allowing users to evaluate peptide antifungal efficacy and safety simultaneously. This streamlined the early stage screening pipeline for peptide drug development and effectively exploited the limited resources.

## Materials and Methods

### Data Collection and Usage

We collected peptide sequences annotated with antifungal activity from five publicly available databases: CAMP,^17^ DRAMP,^18^ YADAMP,^19^ SATPdb,^20^ and DBAASP.^21^ Duplicated sequences and sequences containing unusual amino acid residues (“B”, “Z”, “U”, “J”, “O”, “X”, “i”, “n”, and “–”) were filtered out to conform to the embedding approaches. Considering the length distribution of AFPs with the embedding models, we excluded outlier sequences not in the length range between 10 and 50. To prevent models trained on obviously similar data, we used CD-HIT with an identity threshold of 0.95 to retain only representatives from each similar AFP group. Finally, we obtained 3,011 AFPs as positive samples (Figure 1). We also prepared a negative data set with the same data to ensure that our binary classification models were not biased by data imbalance. The negative data set comprised both randomly generated sequences and non-AMP peptides. We casually linked 20 essential amino acids to generate random sequences with a length distribution matching the positive data set. Additionally, we collected non-AFP peptides from the UniProtKB/Swiss-Prot database, selecting sequences between 10 and 50 amino acids long that lacked unusual amino acid residues and were not labeled with AMP-related keywords such as “Antimicrobial”, “Antibiotic”, or “Defensin”. Nevertheless, we acknowledge that these peptides selected as negative samples may still exhibit other antimicrobial activities that have not yet been discovered or annotated, including potential antifungal activity. Half were randomly generated, and the other half consisted of UniProt peptides, comprising the 3,011 negative data points. We split the data into an independent test set (20%) and used 10% of the remaining data to validate models during training and hyperparameter tuning. Figures S1 and S2 show the length distribution plots of the training and testing data sets. We constructed negative data sets with length distributions similar to those of the positive data sets to prevent machine-learning models from easily predicting AFPs based on peptide length. Besides, the DS2 data sets from AntiFP^4^ were used as the benchmark data set to ensure a fair comparison of model performance. Briefly, the DS2 data sets consisted of 1,168 AFPs as the positive data set and 1,168 non-AMPs as the negative data set, with an average length of around 55 AA.

**Figure 1:**
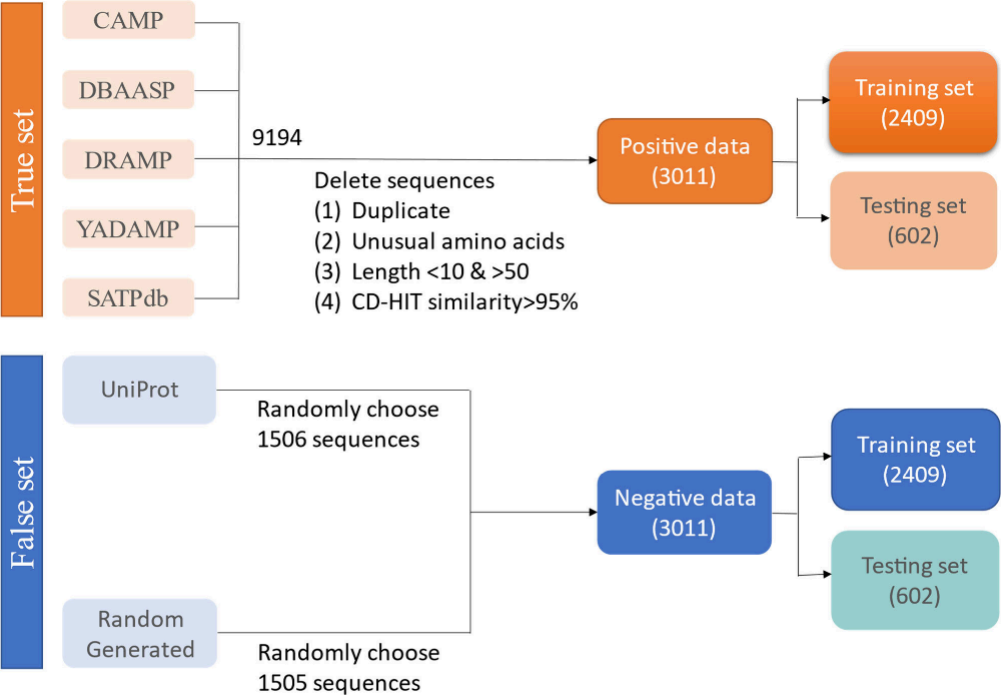
Data collection and usage to build AI4AFP. The positive and negative data amounts were equal to ensure our binary classification models were trained, validated, and tested on balanced data sets.

### Protein Encoding Methods

Protein encoding aimed to convert sequential peptides into computational matrices, which were important for model building. The ideal protein-encoding methods should be able to present protein properties for machine learning algorithms to learn their differences. In this study, we used and compared two protein-encoding methods, Doc2Vec^26^ and PC6.^23^ Doc2Vec was an unsupervised document embedding model transforming peptides into fixed-length vectors. Here, we split every positive AFP data point into 3-mers, meaning that every 3 consecutive amino acids were considered a word. The Doc2Vec model treated each peptide as a sentence and learned its properties to update model weights during training. We implemented Doc2vec using Gensim, an open-source Python library for NLP. We set the “vector_size” parameter in Doc2Vec to 50, transforming peptide sequences into 50-dimensional vectors. The other protein-encoding method was PC6, developed in our previous study,^23^ converting peptide sequences into 2D images based on 6 physicochemical properties of amino acids: hydrophobicity (H1), the volume of side chains (V), polarity (P1), pH at the isoelectric point (pl), the negative of the logarithm of the dissociation constant for the −COOH group (p*K*_a_), and net charge index of the side chain (NCI). The physicochemical property used in PC6 was amino acid-based, meaning each amino acid residue was considered individually rather than averaging across all amino acids for each peptide. Therefore, PC6 had the advantage of simultaneously considering a peptide’s physicochemical properties and the order of its amino acids. Each peptide was zero-padded to a length of 50 and then transformed into corresponding 6 normalized physicochemical property values to form a 50 × 6 matrix in the PC6 protein-encoding method. ProtBert-BFD is a pretrained protein language model based on the BERT (Bidirectional Encoder Representations from Transformers) architecture, specifically developed for comprehensive analysis and feature extraction from protein sequences. The model is trained on the Big Fantastic Database (BFD), which comprises approximately 2.1 billion protein sequences. This large-scale training enables ProtBert-BFD to effectively capture complex sequence dependencies, evolutionary patterns, and functional properties, making it highly suitable for various downstream bioinformatics tasks such as protein classification, structure prediction, and function annotation.

### Machine Learning Algorithms Used in This Study

This study utilized Random Forest (RF), Support Vector Machine (SVM), and deep learning methodologies to build predictive models. We employed Scikit-learn,^27^ a Python package for predictive data analysis, to implement the RF and SVM models, using the default parameters for training. The deep learning model was implemented using TensorFlow^28^ with its high-level API, Keras.

The deep learning model was structured as a single-layer Convolutional Neural Network (CNN). The CNN layer extracted features from encoded peptide sequences, which were then flattened and passed through a fully connected neural network. The final output was a single value, transformed into a probability between 0 and 1 using a sigmoid activation function. To optimize model performance, we fine-tuned key hyperparameters, including the number of CNN layers, a learning rate of 0.0002, a dropout rate of 0.5, and a batch size of 256. After optimization, the final architecture featured a single CNN layer with this configuration, effectively processing peptide sequence data and generating accurate predictions.

### Classifying Antifungal Peptides Using a Fine-Tuned BERT Model

We fine-tuned a BERT model for antifungal peptide classification using protein sequences encoded by ProtBert-BFD. The data set consisted of 3,011 positive and 3,011 negative sequences, which were converted into text format. Each sequence was tokenized and transformed into fixed-length input IDs and attention masks, padded to 50 (Figure S1). The data set was split into 90% training and 10% validation to optimize model performance with a similar length distribution (Figure S2). During fine-tuning, the model parameters were updated for one epoch, with loss and accuracy tracked throughout the training iterations to improve classification accuracy. After training, we assessed the model’s performance on the validation set and generated final predictions on the test set using 10-fold cross-validation. We computed the confusion matrix and ROC curve to evaluate the model’s effectiveness and identify the optimal classification threshold that maximizes performance. Lastly, the trained model was saved in a designated directory for future enhancements.

### Models Ensembling

To enhance predictive performance, we implemented an ensemble strategy that combined multiple models, each trained with different protein-encoding methods and machine-learning algorithms (Figure 2). This approach involved three key steps:

**Figure 2:**
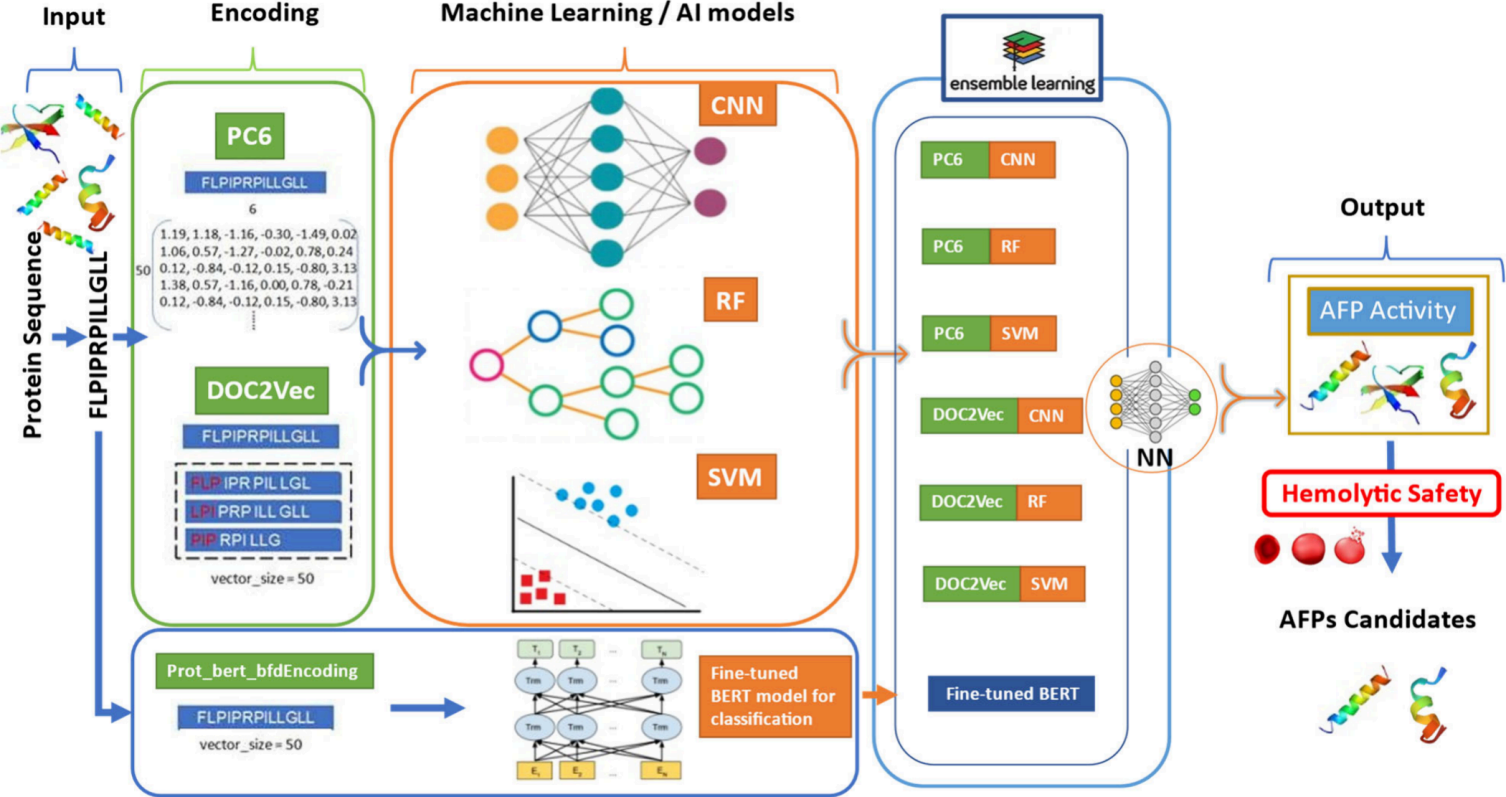
Overview of the AI4AFP model pipeline: Protein sequences are encoded using PC6, Doc2Vec, and Prot_bert_bfd. These embeddings are processed by machine learning models (CNN, RF, SVM) and a fine-tuned BERT classifier. Predictions are integrated via ensemble learning to determine AFP activity and then checked against hemolytic safety in our ensemble-based hemolysis prediction model.

1. Feature Extraction: Protein sequences were encoded using various methods, including PC6, Doc2Vec, and ProtBERT-BFD, to transform them into feature vectors, denoted as *X*_*PC*__6_, *X*_*Doc*_ _2*Vec*_, and *X*_*ProtBERT*_. These features capture diverse sequence characteristics:

1 $$X_{encoded}=f_{encoding}\left(S\right),S\in \mathrm{protein sequences}$$

where *f*_*encoding*_ represents the specific encoding method.

2. Base Model Training: Multiple base classifiers, such as CNN, SVM, RF, and fine-tuned BERT, were trained on these features using 10-fold cross-validation, which ensured robust individual model performance. The output of each base model is

2 $${{ŷ}_{i}}^{\left(k\right)}=h^{\left(k\right)}\left(X_{encoded}\right),i=1,2,...,N$$

where *h* ^(*k*)^ represents the *k*th base model, and *ŷ*_*i*_^(*k*)^ is the prediction for the *i*th sample.

3. Ensemble Meta-Learner: The predictions from the base models were aggregated into stacked features, which were then used as inputs for a neural network meta-learner. This meta-learner captured the relationships between the base models to generate the final ensemble output.

$$Z=\left[\begin{array}{lll} {ŷ}_{1}^{\left(1\right)} & \cdots & {ŷ}_{1}^{\left(K\right)}\\ \vdots & \ddots & \vdots \\ {ŷ}_{N}^{\left(1\right)} & \cdots & {ŷ}_{N}^{\left(K\right)} \end{array}\right],Z\in R^{N\times K}$$ 3

This stacked matrix *Z* was used as input to train a neural network meta-learner.

*ŷ*_*final*_ = *g*(*Z*; θ), where *g* is the neural network meta-learner and θ represents the parameters of a model, such as weights and biases. These parameters are adjusted during training to minimize the error or loss and improve the model’s predictive performance. The meta-learner captures relationships between base models to produce the final ensemble prediction *ŷ*_*final*_. Pseudocode outlining this stacking ensemble model, found in Table S1, integrates three distinct protein-encoding strategies (PC6, Doc2Vec, ProtBERT-BFD) with multiple classifiers (CNN, RF, SVM, fine-tuned BERT), followed by a neural-network meta-learner to improve antifungal peptide classification performance.

### Implementation of the Hemolysis Prediction Model

We obtained a data set comprising 2,984 peptide sequences, with lengths ranging from 15 to 50 residues, and their corresponding hemolysis percentages at the applied concentrations from the DBAASP database.^21^ To enhance model generalizability, redundant sequences with ≥90% similarity were removed.

Each peptide was encoded into a 350-dimensional feature vector by applying the PC6 method, where six physicochemical property values represent each amino acid. A normalized concentration value was appended to each residue, resulting in a 7-dimensional representation per amino acid. Sequences were padded to a uniform length of 50 residues to ensure consistent input size.

We designed five independent binary classifiers with thresholds set at 5%, 10%, 20%, 30%, and 40% to capture the continuous nature of hemolytic risk while preserving interpretability. Each model determines whether a given peptide at a given concentration exceeds the specified hemolysis threshold, enabling multilevel hemolysis estimation based on the classifier outputs.

An ensemble of six machine learning algorithms, including Support Vector Machine (SVM), Random Forest (RF), Multi-Layer Perceptron (MLP), k-Nearest Neighbors (KNN), XGBoost, and AdaBoost, was implemented using soft voting. We systematically explored model combinations to identify the most robust configuration for predicting hemolytic potential across varying thresholds.

### Model Evaluation Metrics

We evaluated model performance using accuracy, precision, sensitivity, specificity, and Matthews Correlation Coefficient (MCC). The following are the equations of model evaluation metrics:

4 $$\mathrm{accuracy}=\frac{TP+TN}{TP+FP+TN+FN}$$

5 $$\mathrm{precision}=\frac{TP}{TP+FP}$$

6 $$\mathrm{sensitivity}=\frac{TP}{TP+FN}$$

7 $$\mathrm{specificity}=\frac{TN}{TN+FP}$$

8 $$MCC=\frac{\left(TP\times TN\right)-\left(FP\times FN\right)}{\sqrt{\left(TP+FP\right)\left(TP+FN\right)\left(TN+FP\right)\left(TN+FN\right)}}$$

where TP represents the true positive predictions, TN represents the true negative predictions, FP represents the false positive predictions, and FN represents the false negative predictions. The Matthews Correlation Coefficient (MCC) is a score that measures the accuracy of a model in classifying two groups. It considers all four outcomes: correct positive and negative predictions, as well as the mistakes. Unlike accuracy, MCC remains reliable even when one group is significantly larger than the other. MCC gives a fair and balanced way to compare our model’s performance.

### Synthesis of Selected Peptides

We used a previously published GAN model to design new peptides,^29^ filtered these with our AFP prediction model, and selected 11 with high predicted antifungal activity for synthesis. Mission Biotech (Taiwan) produced peptides with purity exceeding 95% via solid-phase synthesis and provided mass spectrometry and HPLC data. Peptides were stored as powders at −80 °C and dissolved in sterile water before use.

### Evaluation of Minimal Inhibitory Concentrations (MICs) and Minimal Fungicidal Concentration (MFC)

The minimal inhibitory concentrations (MICs) were determined according to the CLSI protocol M27-A3 and M38-A2. For preparation of the inocula, strains were cultured in 3 mL of YPD (1% yeast extract, 2% peptone, 2% dextrose) broth overnight at 30 °C, washed twice, and resuspended in distilled water. A hemocytometer was used to calculate the concentration of each inoculum. Then it was diluted to the recommended concentrations with RPMI 1640 medium (10.4 g of RPMI 1640 powder, 34.5 g of MOPS [3-(*N*-morph­olino)­prop­ane­sulf­onic acid], 2% dextrose, in 1 L of distilled water, with pH adjusted to 7.0 with NaOH). Peptides were diluted serially 2-fold with RPMI 1640 medium, and 100 μL of each peptide was added to a 96-well plate, followed by 100 μL of the inoculum. The final concentrations of peptides ranged from 0.125 to 64 μg/mL, and the final concentrations of the inocula were 1.25 × 103 cells/mL. Plates were incubated at 35 °C for 48 h, and the MIC was defined as the lowest concentration at which there was no visible colony. The minimal fungicidal concentration (MFC) was determined following the MIC test. For each strain, 3 μL of wells containing the peptide at concentrations from 0.5 MIC (positive control) to the highest concentration (64 μg/mL) were thoroughly pipetted and subcultured onto fresh YPD agar plates. The plates were incubated at 30 °C for 48 h, and the MFC was determined as the drug concentration at which no colonies formed.

### Hemolysis Assay

To evaluate the hemolytic activity of the peptides, a spectroscopic assay was performed using defibrinated sheep blood (Taiwan Prepared Media). The red blood cells (RBCs) were isolated by centrifugation (3000 × g for 10 min at 4 °C), washed three times with PBS, and resuspended to a final concentration of 8 × 10^9^ cells/mL. A 20 μL volume of the RBC suspension was added to 180 μL of peptides (diluted across a specific concentration range) in a 96-well microplate. Following a 30 min incubation at 37 °C without agitation, the plate was centrifuged at 300 × g for 10 min (Eppendorf 5810R). The resulting supernatants (100 μL) were transferred to a new microplate, and hemoglobin release was quantified by measuring absorbance at 540 nm using a microplate reader. Experiments were conducted in triplicate, utilizing distilled water as a positive control (100% lysis) and PBS as a negative control (0% lysis). The hemolysis rate was calculated according to the following formula:^30^

9 $$\mathrm{hemolysis rate}\;\left(\%\right)=\frac{OD_{test}-OD_{negative\;control}}{OD_{positive\;control}-OD_{negative\;control}}\times 100\%$$

The Minimum Hemolytic Concentration (MHC_10_) was defined as the lowest peptide concentration resulting in 10% hemolysis relative to the positive control.

## Results and Discussion

### Model Performances Based on DS2 Data Sets

We used the publicly available DS2 data set from Antifp for training and testing, ensuring fair comparisons with existing predictors by maintaining consistent data set sizes. This approach enabled us to evaluate model architectures rigorously. Following prior studies, we assessed model performance using accuracy (Acc), sensitivity (Sen), specificity (Spe), and Matthews correlation coefficient (MCC). Table 1 presents the performance of our models trained and tested on the DS2 data set, alongside results from Antifp,^4^ AFPDeep,^22^ and DeepAFP.^6^ Precision was not included in the comparison because it was not reported in the referenced studies.

**Table 1:** Performance Comparison of AI4AFP Models with Existing Predictors Using DS2 Training and Testing Data Sets^a^

Encoding Method/AI Model	Acc	Sen	Spe	MCC
Models Used in This Study				
PC6/RF	0.91	0.87	0.96	0.83
PC6/SVM	0.91	0.88	0.94	0.82
PC6/CNN	0.90	0.91	0.89	0.79
Doc2vec/RF	0.84	0.88	0.79	0.67
Doc2vec/SVM	0.83	0.86	0.82	0.68
Doc2vec/CNN	0.80	0.83	0.78	0.61
Prot_bert_bfd/BERT	0.93	0.90	0.95	0.85
Ensemble_seven_model	**0.95**	**0.92**	**0.97**	**0.89**
Existing Approaches				
Hybrid features (cluster profile+physiochemical property)/DNN (Antifp, 2018)	0.90	0.91	0.90	0.81
Embedding layer/CNN+LSTM (AFPDeep, 2019)	0.89	**0.92**	0.86	0.77
Hybrid features (Bert+Binary profile+BLOSUM62)/Transformer-based model+CNN-BiLSTM (DeepAFP, 2023)	**0.96**	**0.95**	**0.97**	**0.93**

a The metrics include accuracy (Acc), sensitivity (Sen), specificity (Spe), and Matthews correlation coefficient (MCC). The highest values for each metric are bolded, while the second-best values are bolded and underlined (e.g., 0.95 for accuracy and 0.89 for MCC). The results highlight that our ensemble model achieves competitive performance, ranking among the top models and demonstrating its effectiveness in antifungal peptide prediction.

Among our PC6-based models, the PC6-RF achieved the highest MCC (0.83), surpassing Antifp’s MCC of 0.81. Additionally, our BERT-based model (Prot_bert_bfd/BERT) demonstrated the strongest individual performance, with an MCC of 0.85. Notably, our ensemble model (Ensemble_seven_model) further improved upon these results, achieving accuracy (0.95), sensitivity (0.92), specificity (0.97), and MCC (0.89). These findings underscore the effectiveness of integrating multiple encoding methods and models for enhanced predictive capability. Its performance was close to DeepAFP, which achieved the best overall performance on DS2, with an accuracy of 0.96 and MCC of 0.93.

DeepAFP, a hybrid transformer-based model that combines BERT, binary profile, and BLOSUM62 features, achieved the highest overall performance. This suggests that hybrid feature representations and transformer architectures can further enhance peptide sequence prediction. Additionally, we observed that our Doc2Vec-encoded models exhibited higher specificity but lower sensitivity, suggesting that Doc2Vec models may require a larger data set for effective pretraining, and the DS2 data set may not have been sufficient compared to our collected data sets.

### Model Performances Based on Our Data Sets

To develop AI4AFP, we trained and evaluated several models using different encoding methods and machine learning algorithms on the data sets we collected. We set aside 10% of our data sets for testing, and used the remaining 90% for 10-fold cross-validation to assess model stability. The model demonstrates consistently high performance across all folds, with Accuracy and F1-score values averaging around 94% and MCC values ranging from 0.86 to 0.88, indicating robust and balanced predictive capability (Table S2). AFPDeep did not provide their predictor and model to users, so we only submitted our testing data set to the Antifp web server for their prediction results. The performance of our models was evaluated using five statistical metrics: accuracy, precision, sensitivity, specificity, and MCC. The results are summarized in Table 2. Notably, we observed that the Doc2vec encoding method performed better than expected, despite its poor performance in previous experiments on the DS2 data sets.

**Table 2:** Prediction Performance of Different Models and Encoding Methods on the Collected Data Set^a^

Encoding Method	Model	Acc	Pre	Sen	Spe	MCC
Testing (20% of the collected data set)						
Doc2Vec+PC6+BERT	Ensemble model	**0.94**	**0.95**	**0.94**	**0.95**	**0.89**
Doc2Vec	RF	0.91	0.89	**0.94**	0.88	0.82
	SVM	**0.93**	**0.95**	**0.91**	**0.95**	**0.86**
	CNN	0.92	0.93	0.91	0.93	0.84
PC6	RF	0.91	**0.96**	0.86	**0.96**	0.83
	SVM	0.90	0.92	0.87	0.92	0.80
	CNN	0.87	0.87	0.88	0.87	0.74
Prot_bert_bfd	BERT	0.91	0.93	0.89	0.93	0.82
Antifp (2018)	DNN	0.50	0.52	0.10	0.90	0.01
DeepAFP (2023)	CNN+BiLSTM	0.47	0.45	0.23	0.71	–0.058

a The evaluation metrics include accuracy (Acc), precision (Pre), sensitivity (Sen), specificity (Spe), and Matthews correlation coefficient (MCC). The highest values for each metric are in bold, while the second-best values are in bold and underlined. The results show that the ensemble model, incorporating embedding methods (Doc2Vec, PC6, and BERT), achieves the best overall performance across multiple metrics, demonstrating its effectiveness in antifungal peptide prediction.

To summarize, the combinations of CNN and SVM with the Doc2vec encoding method, with MCCs of 0.85 and 0.88, outperformed the other combinations. Both combinations got an average MCC of 0.85 in 10-fold cross-validation, outperforming other combinations. In addition, the prediction results of Antifp were considerably poor when tested by our collected data sets. That was because the data sets they used to train models were collected years ago and lacked enough AFPs to represent the entire AFP population.

### Ensemble Strategy Enhances Predictive Power of AI4AFP

Due to the comparable performance of multiple models using different protein-encoding methods and machine learning algorithms, selecting the best model for developing AI4AFP proved challenging. We applied ensemble techniques to integrate multiple models and improve predictive performance.

Table 2 presents the results of different models tested on 20% of the collected data set. Among individual models, Doc2Vec-SVM achieved the highest MCC (0.86), followed by PC6-RF (0.83) and BERT (0.82). This indicates that different encoding methods may contribute unique strengths to classification performance.

We employed a stacking ensemble strategy to improve further prediction accuracy, where outputs from multiple models were combined and fed into a neural network-based meta-learner (Figure 2). The ensemble model incorporating Doc2Vec, PC6, and BERT features demonstrated the highest performance, achieving an MCC of 0.89, an accuracy of 0.94, and a precision of 0.95. This result surpasses all individual models, highlighting the effectiveness of integrating multiple encoding approaches.

DeepAFP, despite ranking highest in Table 1, performed poorly on the data set we collected (MCC = −0.058, Acc = 0.47), indicating that its CNN+BiLSTM architecture may not generalize well beyond its original training data. Similarly, the Antifp^4^ model demonstrated weak performance (MCC = 0.01, Accuracy = 0.50), underscoring the need for diverse encoding strategies and ensemble learning in robust antimicrobial peptide prediction. One likely reason for DeepAFP’s limited generalizability is its negative data set composition; unlike our strict approach, which excludes peptides with AMP-related keywords, its negative data set (e.g., Antifp DS2) may include unfiltered nonantifungal peptides or AMPs with other activities, causing the model to learn nonspecific features. Moreover, DeepAFP and Antifp were trained on the DS2 data set, in which approximately half of the sequences are longer than 50 amino acids. In our data set, the sequence lengths range from 10 to 50 amino acids, which poses additional challenges for model training and may further contribute to the poor performance observed in both models. In contrast, our more rigorous negative data set construction yields a more discriminative model. Overall, our seven-model ensemble achieved an AUC of 0.98, outperforming the other models and demonstrating the value of ensemble strategies (Figure 3). The final AI4AFP system was developed using this ensemble approach, and the pseudocode for the stacking ensemble model for antifungal peptide classification is provided in Table S1.

**Figure 3:**
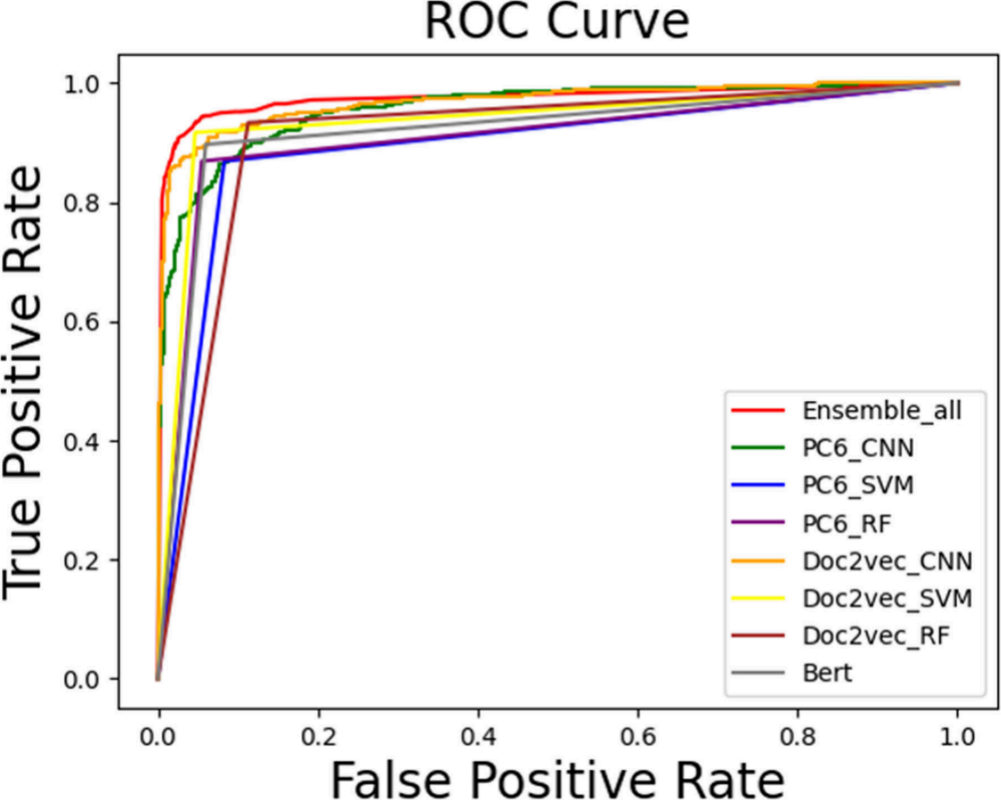
Receiver operating characteristic (ROC) curves comparing model performance across feature representations and classifiers. The ROC curves display the performance of various models using different feature representations (PC6 and Doc2Vec) and classification algorithms (CNN, SVM, RF, and ensemble). The Area Under the Curve (AUC) values indicate the classification accuracy, with the Ensemble_all model achieving the highest AUC of 0.98.

### Hemolysis Prediction

The data set used for hemolytic prediction came from DBAASP.^21^ The labels depend on the threshold we are examining. For example, if a sequence kills 5% of erythrocytes, it will be labeled as hemolytic at a threshold 5% and nonhemolytic otherwise. Table S3, the data set used for hemolysis prediction, includes each row with the counts of positive and negative amino acid sequences for different hemolysis percentages: 0%, 10%, 20%, 30%, and 40%, etc.

Peptide sequences with associated concentration data are first encoded using the PC6 method, which incorporates six physicochemical properties plus one concentration feature (6 + 1 encoding, with a fixed sequence length of 50). These encoded representations serve as inputs to an ensemble learning framework comprising multiple machine learning models, including Random Forest (RF), k-Nearest Neighbors (kNN), Support Vector Machine (SVM), Multi-Layer Perceptron (MLP), and XGBoost. The ensemble model is trained on experimentally validated hemolysis data to predict whether a given peptide induces hemolysis beyond a certain threshold (Figure 4).

**Figure 4:**
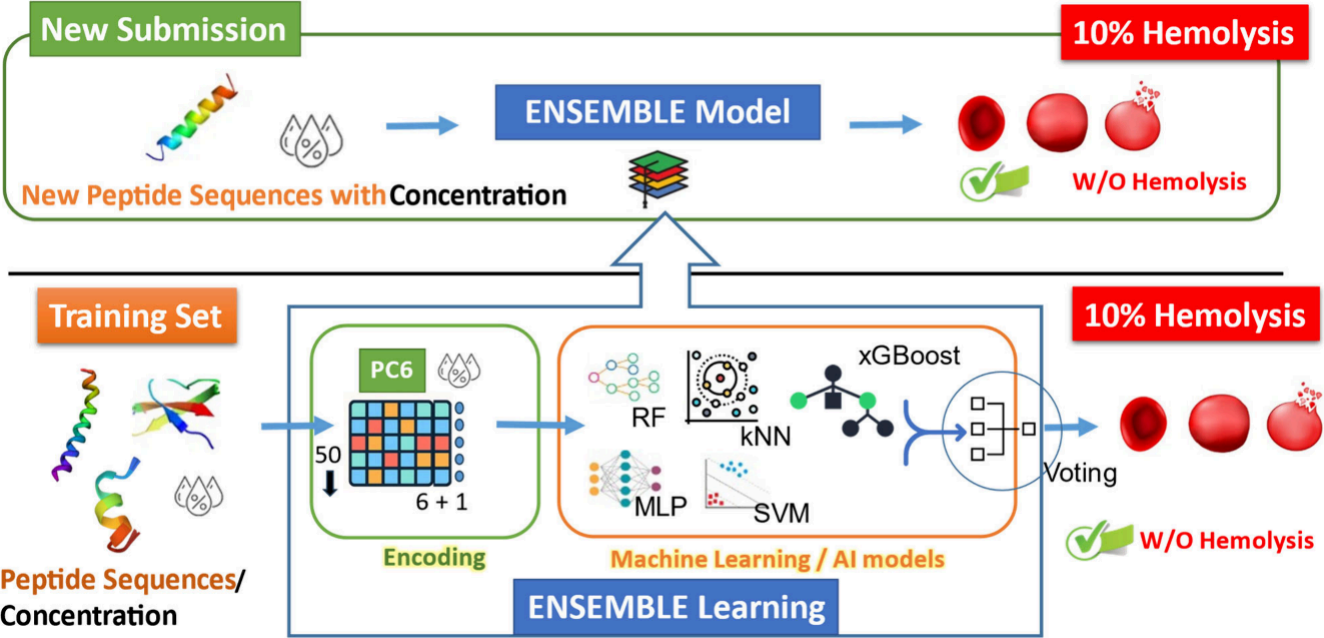
Schematic workflow of the ensemble-based hemolysis prediction model. Peptide sequences and their corresponding concentration data are first transformed using the PC6 encoding method, which combines six physicochemical properties with one concentration feature (resulting in a 6 + 1 scheme, with sequences standardized to a length of 50). These encoded vectors are then fed into an ensemble learning framework composed of diverse machine learning algorithms, including Random Forest (RF), k-Nearest Neighbors (kNN), Support Vector Machine (SVM), Multi-Layer Perceptron (MLP), and XGBoost. The model is trained on experimentally verified hemolysis data to predict whether a peptide will likely exceed a predefined hemolysis threshold. Peptides predicted to have low hemolytic potential (≤10%) while retaining antifungal activity are prioritized for subsequent validation in wet-lab experiments.

While the HAPPENN^10^ model introduced a valuable effort to categorize hemolytic peptides based on experimentally derived data, it still relied on simplified, threshold-based binary classification. According to its original publication, the classification standards presented in Table 1 were derived from 3,738 peptide sequences and their corresponding hemolytic activities collected from the DBAASP and Hemolytik databases. Although these thresholds were grounded in experimental observations, the study itself acknowledged several limitations: variability in assay conditions across studies (e.g., red blood cell source, incubation time, temperature), inconsistent definitions of minimum hemolytic concentration (MHC), and a lack of transparency regarding how threshold values were selected. Notably, definitions of MHC varied widely—from concentrations causing 5%, 10%, or 50% hemolysis to the maximum nonhemolytic dose—making it difficult to apply a consistent standard across all data. Furthermore, reducing hemolytic behavior into a binary classification inevitably obscures the dose-dependent nature of toxicity, which exists on a continuous spectrum.

In contrast, our hemolysis prediction model is the first to explicitly integrate peptide sequence and its corresponding concentration as continuous inputs, enabling a more biologically grounded prediction of hemolytic potential. Rather than imposing arbitrary thresholds, we use actual experimental hemolysis percentages as training labels, preserving the quantitative relationship between dosage and toxicity. This design allows AI4AFP to reflect better real-world scenarios in which peptide safety depends on concentration. Additionally, this structure allows the model to flexibly support stratified classification under different safety thresholds (e.g., 5%, 10%, 20% hemolysis), as demonstrated in Table S4. By incorporating both the mechanistic and contextual dimensions of hemolytic activity, our model offers improved interpretability, biological relevance, and practical applicability in early stage peptide screening and optimization.

Although we have trained separate models for various hemolysis levels (5%, 10%, 20%, 30%, and 40%), only the 10% threshold is shown here for illustration and simplicity. Peptides predicted to be nonhemolytic (≤10%) are prioritized for further development as safe antifungal candidates (Figure 4). Comparison of accuracy and precision between our ensemble model (10-fold cross-validation) and HAPPENN (original validation set) across hemolysis thresholds from 5% to 40%. Our method performs better in both metrics, with higher average values across all thresholds (Table S4).

### Model Prediction Reflects General Antifungal Potential Rather than Species-Specific Potency

To evaluate the generalizability of the proposed AFP prediction model, representative high-scoring peptides were experimentally tested against three phylogenetically and clinically distinct fungal species, including *Candida albicans* SC5314 (Table 3), *Candida glabrata* CBS138 (Table S5), and *Cryptococcus neoformans* H99 (Table S6). In the case of *C. albicans*, 3 out of 11 top-scoring peptides exhibited measurable antifungal activity, indicating an apparent false positive rate when evaluated against this single species. We acknowledge this observation and emphasize that the model was developed as a binary classifier to estimate general antifungal potential rather than species-specific efficacy.

**Table 3:** Antifungal Activity of Model-Predicted AFP Candidates against *Candida albicans* SC5314^a^

Peptide	AFP Predicted Score	MIC	MFC	Hemolysis Prediction for MIC/MFC	MHC_10_ ^a^
GAN-pep1	0.97	64	>64	Y/Y	40.3
GAN-pep2	0.99	>64	-	N/-	25.7
GAN-pep4	1	>64	-	N/-	12.7
GAN-pep5	0.76	>64	-	Y/-	34.0
GAN-pep6	0.81	32	32	Y/Y	34.0
GAN-pep7	0.72	64	64	Y/Y	36.5
Pep-9m_g	0.99	4	4	Y/Y	2.2
AMP_23_gag_20	1	64	64	N/N	11.6
AFP_23_gag_18	1	>64	-	N/-	ND^b^
AVP_23_gag_23	0.99	>64	-	Y/-	60.6
ACP_23_gag_24	1	16	16	Y/Y	ND

a The predicted score reflects the general antifungal potential inferred from peptide-sequence features and is not intended to represent species-specific potency. MIC and MFC values indicate experimentally measured inhibitory activity under the tested conditions. Hemolysis prediction and the experimentally determined minimum concentration inducing 10% hemolysis (MHC_10_) are provided to contextualize concentration-dependent toxicity and were not used to assess prediction accuracy.

b 10% minimal hemolysis concentration (MHC_10_) was defined as the lowest concentration that induced 10% hemolysis.

c ND: not detected.

Consistent with the model design, predicted antifungal scores reflect general antifungal potential rather than species-specific potency. Accordingly, peptides with high predicted scores exhibited heterogeneous inhibitory profiles across different fungal species, highlighting the context-dependent nature of antifungal activity.

Notably, several peptides that showed limited activity against *C. albicans* demonstrated pronounced inhibitory effects against *C. glabrata* or *C. neoformans*, underscoring that the absence of activity against a single species does not imply incorrect prediction. Instead, these results support the model’s ability to prioritize peptides with broad antifungal potential that may manifest selectively depending on fungal biology.

In parallel, hemolytic risk was evaluated using a complementary strategy combining prediction and experimental validation. Hemolysis predictions were assessed at the experimentally determined MIC or MFC for each peptide–fungus combination, reflecting concentration-dependent toxicity. In addition, the minimum concentration inducing 10% hemolysis (MHC_10_) was experimentally determined to provide an empirical safety threshold. Together, these analyses indicate that hemolytic effects are governed by both peptide sequence and effective antifungal concentration, and that several peptides retained antifungal activity under conditions associated with limited hemolysis.

Collectively, the multispecies validation demonstrates that the proposed model functions as a general AFP prioritization tool, capable of enriching for peptides with antifungal potential across diverse fungal contexts. At the same time, potency and toxicity should be interpreted in a species- and concentration-dependent manner rather than as direct reflections of prediction score magnitude.

### Future Directions: Toward Safer and Smarter AFP Discovery

o further improve the predictive power of AI4AFP, future efforts should consider integrating more advanced sequence encoding techniques, including emerging protein and peptide large language models such as ProtT5,^31^ ESM-2,^32^ peptideBERT,^33^ and ProtGPT-2.^34^ Enhancing model inputs with additional structural features and biologically relevant properties, and expanding the training data set through careful negative sample selection and the generation of novel peptide candidates, is expected to increase model robustness substantially. Importantly, the current AI4AFP framework is designed as a general antifungal peptide (AFP) prioritization tool, aiming to capture broad antifungal potential rather than species-specific potency. As antifungal activity is inherently context- and species-dependent, future development of species-specific classifiers may further refine prediction accuracy when targeting particular pathogens such as *Candida albicans*, *C. glabrata*, or *C. neoformans*. Within this framework, transfer learning or fine-tuning strategies could be employed to adapt the general AFP model to specific fungal species, provided that sufficient species-resolved AFP data become available.

Wet-lab validation remains an essential component of AFP discovery, not only to confirm antifungal efficacy but also to assess safety profiles. In particular, hemolysis and cytotoxicity assays should be interpreted in a concentration-dependent manner, as hemolytic risk is governed by both peptide sequence features and the effective antifungal concentration (e.g., MIC or MFC). Consistent with this view, experimental determination of the minimum concentration inducing 10% hemolysis (MHC_10_) provides a practical safety reference rather than an absolute toxicity threshold, underscoring the importance of contextualizing safety alongside antifungal activity.

We are currently developing an enhanced framework that integrates a sequence-based hemolysis predictor with a functional antifungal activity classifier to enable a more comprehensive evaluation of peptide candidates. This integrated system will be implemented within AI4AFP using a reinforcement learning (RL) strategy that simultaneously promotes high antifungal potential and low hemolytic risk. By explicitly optimizing these dual objectives, the framework aims to accelerate the discovery of AFPs that are both effective and biologically safer. This RL-enhanced approach builds upon our prior work involving GAN-based peptide generation,^29^ where several generated antimicrobial peptides successfully disrupted the membranes of diverse bacterial pathogens, including *E. coli* and MRSA. Encouraged by these results, we are now extending this strategy to AFP discovery against fungal pathogens, including *C. albicans* (SC5314), *C. glabrata* (CBS138), and *C. neoformans* (H99). By coupling general AFP prediction, species-aware refinement, and concentration-dependent safety assessment within an RL framework, AI4AFP offers a scalable pathway toward the rational design of next-generation antifungal peptides with improved efficacy and safety profiles.

## Conclusion

We developed AI4AFP, an ensemble-based framework integrating multiple peptide encoding strategies (PC6, Doc2Vec, and ProtBERT-BFD) with diverse machine learning models, achieving robust antifungal peptide (AFP) prediction performance on an independent validation set. Importantly, the predicted AFP score reflects general antifungal potential rather than species-specific potency, consistent with the observed context-dependent activity across different fungal species. To complement efficacy prediction, we introduced a hemolysis classifier that models toxicity as a dose-dependent property by jointly considering peptide sequence and applied concentration, enabling flexible safety thresholds beyond binary classification. All components are implemented in a user-friendly web server, allowing integrated evaluation of antifungal activity and hemolytic risk. Future extensions incorporating species-aware fine-tuning and reinforcement-learning-based optimization are expected further to accelerate the discovery of safer and more effective AFPs. All models were implemented in a user-friendly web server, AI4AFP (Figure 5), enabling comprehensive evaluation of antifungal peptides in terms of efficacy and safety.

**Figure 5:**
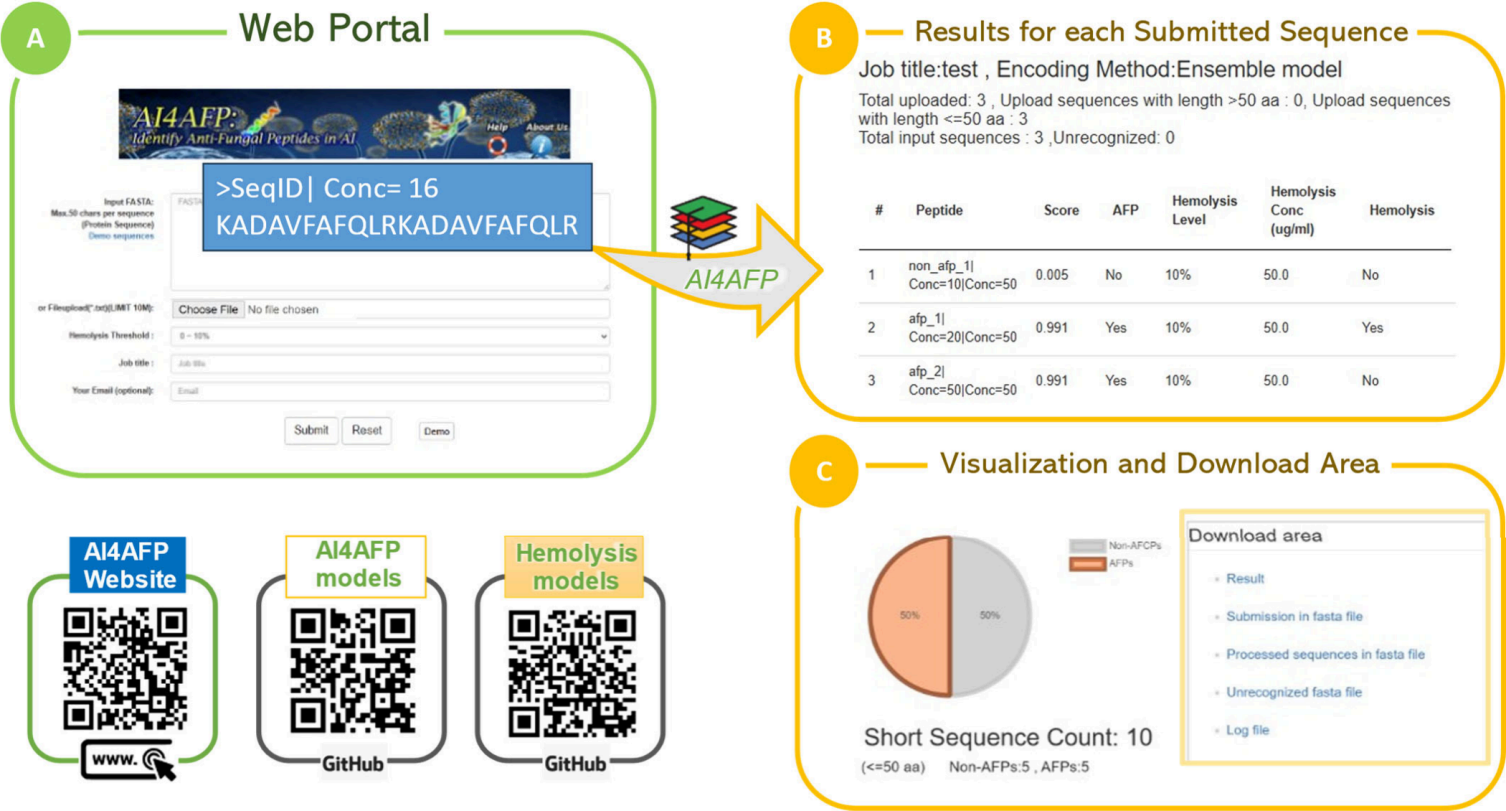
Summary of the AI4AFP web portal for simultaneous prediction of antifungal activity and hemolytic safety. (A) Users can submit peptide sequences in FASTA format (up to 50 amino acids per sequence) or upload a .txt file. Each input may include a concentration value (e.g., Conc = 16, meaning 16 μg/mL) for hemolysis prediction. Optional fields like job title and email are available for personalized task tracking. (B) For each submitted sequence, the ensemble model outputs a classification score, AFP prediction, hemolysis level, the concentration used for evaluation, and whether the peptide is considered hemolytic. This integrated result allows simultaneous interpretation of antifungal potential and cytotoxicity risk. (C) Prediction results are summarized with interactive visualizations (e.g., pie charts showing AFP distribution) and downloadable files, including processed input, result reports, and unrecognized sequences. This feature facilitates the interpretation of results and batch analysis.

## Supplementary Material



## Data Availability

AI4AFP is freely available at https://axp.iis.sinica.edu.tw/AI4AFP/. The source code and data sets for predicting antifungal peptide (AFP) activity and assessing hemolytic safety are available at https://github.com/lsbnb/AI4AFP and https://github.com/ulin0729/peptide-hemolysis-self. The peptide sequences identified in this study are currently subject to pending patent applications and therefore cannot be fully disclosed at this stage. Sequence-level information will be made available upon patent publication or as permitted by intellectual property regulations. All functional assay results, statistical analyses, and model evaluation data necessary to support the conclusions of this study are provided in the manuscript and its Supporting Information.

## Notes

Overview of data set attributes, model framework outline, validation performance metrics, data set used for hemolysis prediction, comparative advancements relative to established methods, and experimental assessment of predicted antifungal peptides across diverse fungal species (PDF)
