# Supplementary material for: Harnessing Sequence Embedding and Ensemble Learning to Identify Antifungal Peptides with Low Hemolytic Risk
Source: ACS Omega. 2026 Apr 27;11(18):26870–80. doi: 10.1021/acsomega.6c00049 (PMC13177248; doi:10.1021/acsomega.6c00049)
Supplement: Supplementary file 1 [file ao6c00049_si_001.pdf]

Supporting Information

For

Harnessing Sequence Embedding and Ensemble Learning to Identify Antifungal Peptides with Low Hemolytic Risk

Chung-Yen Lin[a,b,c§],Wen-Chih Cheng[a§], U-Lin Chen[d], Tzu-Tang Lin[e], Li-Hang Hsu[f], Yang-Hsin Shih[g], I-Hsuan Lu[a], Ying-Lien Chen[f], Shu-Hwa Chen[h*]

[a] Institute of Information Science, Academia Sinica, TAIWAN., [b] Institute of Fishery Sciences, College of Life Science, National Taiwan University, Taipei, TAIWAN [c] Genome and Systems Biology Degree Program, National Taiwan University, Taipei, TAIWAN [d] Data Science Program, National Taiwan University, Taipei, TAIWAN [e] College of Pharmacy, University of Florida, United States [f] Department of Plant Pathology and Microbiology, National Taiwan University, Taipei, TAIWAN [g] Department of Agricultural Chemistry, National Taiwan University, No. 1, Sec. 4, Roosevelt Rd., Taipei 10617, TAIWAN [h]TMU Research Center of Cancer Translational Medicine, Taipei Medical University, Taipei, TAIWAN

*Corresponding author: Shu-Hwa Chen (sophia0715@tmu.edu.tw)

§ C.Y.L. and W.C.C. contributed equally to this work

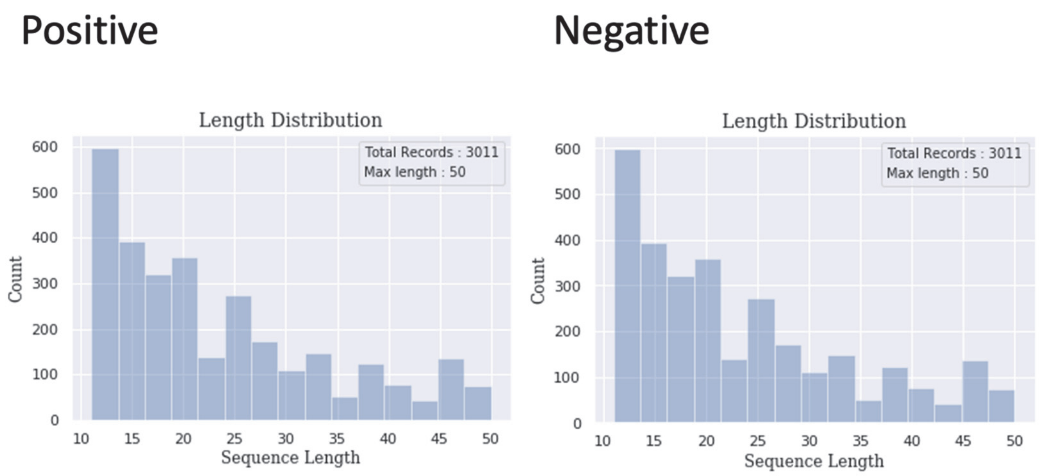

Figure S1. The length distribution plots of the training datasets. We built the negative datasets with length distributions similar to those of the positive datasets to prevent machine learning models from predicting AFPs based on peptide length.

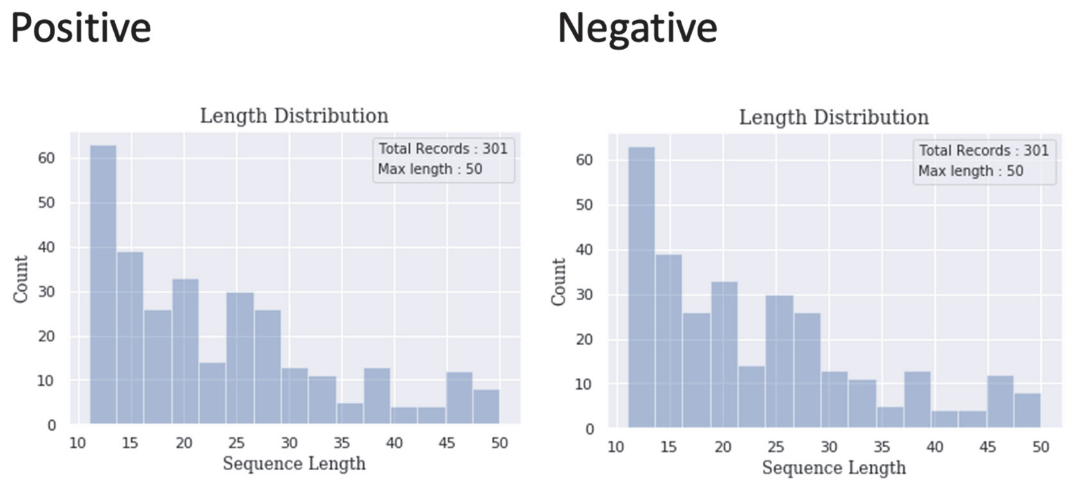

Figure S2. The length distribution plots of the testing datasets. We built the negative datasets with length distributions similar to those of the positive datasets to prevent machine learning models from predicting AFPs based solely on peptide length.

Table S1. Integrated Ensemble Framework Combining Multi-Modal Feature Encodings and Classifiers for Robust Antifungal Peptide Prediction. Pseudocode for Stacking Ensemble Model in Antifungal Peptide Classification. Using a stacking ensemble approach, this workflow integrates multiple protein encoding methods (PC6, Doc2Vec, ProtBERT-BFD) and machine learning models (CNN, RF, SVM, Fine-tuned BERT). A meta-learner (Neural Network) refines predictions, improving AFP classification accuracy through model fusion.

```
BEGIN Stacking_Ensemble_Model

    # Concept:
    # This script uses multiple encoding methods (PC6, Doc2Vec, ProtBERT-BFD) to extract protein sequence features.
    # It then trains multiple base classifiers (CNN, SVM, RF, Fine-Tuned BERT) using K-Fold Cross-Validation.
    # The predictions from these base models are combined into an ensemble model using a neural network.

    # Step 1: Load and Encode Protein Sequence Data
    INPUT: Protein sequences (Positive & Negative)
    DEFINE train, validation, and test data paths

    CONVERT sequences into encoded features using:
        - PC6 Encoding
        - Doc2Vec Encoding
        - ProtBERT-BFD Pre-trained Model (MAX_LEN = 50)

    CONVERT labels to numpy arrays

    # Step 2: Define Base Models
    DEFINE Base_Models = {CNN, Random Forest (RF), SVM, Fine-tuned BERT}

    # Step 3: Train Individual Models Using K-Fold Cross-Validation

    DEFINE K = 10      # Number of folds
    INITIALIZE results storage

    FOR each fold k in 1 to K DO:
        SPLIT data into Training_Set (90%) and Validation_Set (10%)

        ## PC6-Based Models
        TRAIN SVM on PC6 Features → Save as `svm_k.pkl`
        TRAIN Random Forest on PC6 Features → Save as `rf_k.pkl`
        TRAIN CNN on PC6 Features → Save best weights as `cnn_k_best_weights.h5`
        DETERMINE CNN threshold using ROC analysis

        ## Doc2Vec-Based Models
        TRAIN SVM on Doc2Vec Features → Save as `svm_k.pkl`
        TRAIN Random Forest on Doc2Vec Features → Save as `rf_k.pkl`
        TRAIN CNN on Doc2Vec Features → Save best weights as `cnn_k_best_weights.h5`
        DETERMINE CNN threshold using ROC analysis

        ## BERT Model
        TRAIN Fine-tuned BERT using pre-trained `Rostlab/prot_bert_bfd`
        SAVE BERT model predictions

        STORE predictions for ensemble training

    END FOR

    # Step 4: Construct Stacking Feature Matrix (Z)
    DEFINE Matrix Z of size (N_samples, N_models)

    FOR each validation sample:
        STORE:
            - PC6 RF, SVM predictions
            - PC6 CNN binary prediction (thresholded)
```

```
            - Doc2Vec RF, SVM predictions
            - Doc2Vec CNN binary prediction (thresholded)
            - BERT prediction

    # Step 5: Train Meta-Learner (Neural Network)
    DEFINE Meta-Learner as Neural Network (Input: Z)
    TRAIN Meta-Learner using (Z, Y)
    SAVE trained ensemble model

    # Step 6: Final Prediction on Independent Test Data
    FUNCTION Stacking_Predict(X_new):
        CREATE Matrix Z_new of size (N_new_samples, N_models)

        FOR each model f_i in Base_Models DO:
            IF f_i == Fine-tuned BERT THEN:
                Tokenize X_new
                PREDICT probabilities y_pred using Fine-tuned BERT
            ELSE:
                PREDICT probabilities y_pred on X_new

            STORE y_pred in corresponding column of Z_new

        RETURN Meta_Learner_Predict(Z_new) ≥ 0.5    # Apply classification threshold

    END FUNCTION

    # Step 7: Output Results
    OUTPUT:
        - Final ensemble model performance
        - Individual model performances
        - PC6 RF, SVM, CNN scores
        - Doc2Vec RF, SVM, CNN scores
        - BERT model score

END Stacking_Ensemble_Model
```

Table S2. Cross-Validation Performance Metrics of the AFP ensemble Classification Model on our data collection. This table summarizes the 10-fold cross-validation results of the Ensemble model, reporting key performance metrics, including Accuracy, Precision, Sensitivity, Specificity, F1-score, and Matthews Correlation Coefficient (MCC) for each fold. The model demonstrates consistently high performance across all folds, with Accuracy and F1-score values averaging around 94% and MCC values ranging from 0.86 to 0.88, indicating robust, balanced predictive performance.

#fold	Accuracy	Precision	Sensitivity	Specificity	F1-score	MCC
1	0.94	0.94	0.94	0.94	0.94	0.88
2	0.94	0.93	0.94	0.93	0.94	0.87
3	0.94	0.94	0.94	0.94	0.94	0.88
4	0.94	0.94	0.93	0.95	0.94	0.87
5	0.94	0.94	0.93	0.94	0.94	0.88
6	0.94	0.94	0.94	0.94	0.94	0.88
7	0.93	0.92	0.94	0.92	0.93	0.86
8	0.94	0.95	0.93	0.95	0.94	0.88
9	0.94	0.95	0.93	0.95	0.94	0.88
10	0.94	0.95	0.91	0.95	0.94	0.88

Table S3. Dataset used for Hemolysis Prediction. The data came from DBAASP [1]. The labels depend on which threshold we are looking at. For example, if a sequence kills 5% of erythrocytes, it will be labeled as hemolytic at a threshold of 5%; otherwise, non-hemolytic. Each row in the dataset (2984) includes the amino acid sequence, concentration, and hemolysis percentage.

Hemolysis Threshold	Total (Pos+Neg)	Train/Test Pos	Train/Test Neg
5%	2186	874/219	874/219
10%	2782	1112/279	1113/278
20%	2434	973/244	974/243
30%	2246	898/225	898/225
40%	2146	858/215	858/215

Table S4. Improved Performance Over HAPPENN Across Hemolysis Thresholds. Comparison of accuracy and precision between our ensemble model (10-fold cross-validation) and HAPPENN (original validation set) across hemolysis thresholds from 5% to 40%. Our method performs better in both metrics, with higher average values across all thresholds.

	Metrics	5%	10%	20%	30%	40%	Average over thresholds
This study	Accuracy	0.80	0.78	0.77	0.77	0.80	0.78
	Precision	0.81	0.79	0.77	0.77	0.83	0.80
HAPPENN	Accuracy	0.75	0.69	0.67	0.63	0.63	0.67
	Precision	0.30	0.46	0.55	0.56	0.63	0.50

Table S5. Antifungal activity of model-predicted AFP candidates against *Candida glabrata* CBS138. As the AFP prediction model is designed to identify broad antifungal potential rather than strain-specific activity, variations in MIC (μg/ml) and MFC (μg/ml) across fungal species are expected. Hemolysis prediction at the effective antifungal concentration and experimentally measured MHC_{10} (μg/ml) values are included to provide a complementary safety context.

Peptide	*Predicted Anti-fungal ability*	MIC	MFC	*Hemolysis Prediction* of MIC/MFC	MHC_{10}^{a}
**GAN-pep1**	0.97	>64	-	Y/-	40.3
**GAN-pep2**	0.99	>64	-	N/-	25.7
**GAN-pep4**	1	32	32	N/N	12.7
**GAN-pep5**	0.76	>64	-	Y/-	34.0
**GAN-pep6**	0.81	32	>64	Y/Y	34.0
**GAN-pep7**	0.72	>64	-	Y/-	36.5
**Pep-9m_g**	0.99	8	8	Y/Y	2.2
**AMP_23_gag_20**	1	>64	-	N/-	11.6
**AFP_23_gag_18**	1	16	32	N/N	ND[b]
**AVP_23_gag_23**	0.99	>64	-	Y/-	60.6
**ACP_23_gag_24**	1	64	64	Y/Y	ND

[a] 10% minimal hemolysis concentration (MHC10) was defined as the lowest concentration that induced 10% hemolysis.

[b] ND: not detected.

Table S6. Antifungal activity of model-predicted AFP candidates against *Cryptococcus neoformans H99*. Differences in antifungal efficacy among fungal species reflect the context-dependent manifestation of antifungal activity, rather than inaccuracies of the general AFP prediction model. Hemolytic risk was evaluated in a concentration-dependent manner using both MIC/MFC-based prediction (μg/ml) and experimental determination of MHC_{10} (μg/ml).

Peptide	*Predicted Anti-fungal ability*	MIC	MFC	*Hemolysis Prediction* for MIC/MFC	MHC_{10}[a]
**GAN-pep1**	0.97	64	64	Y/Y	40.3
**GAN-pep2**	0.99	64	64	N/N	25.7
**GAN-pep4**	1	16	16	N/N	12.7
**GAN-pep5**	0.76	>64	-	Y/-	34.0
**GAN-pep6**	0.81	16	32	Y/Y	34.0
**GAN-pep7**	0.72	>64	-	Y/-	36.5
**Pep-9m_g**	0.99	*	*	Y/Y	2.2
**AMP_23_gag_20**	1	32	32	N/N	11.6
**AFP_23_gag_18**	1	8	8	N/N	ND[b]
**AVP_23_gag_23**	0.99	64	>64	Y/Y	60.6
**ACP_23_gag_24**	1	16	16	Y/Y	ND

[a] 10% minimal hemolysis concentration (MHC_{10}) was defined as the lowest concentration that induced 10% hemolysis.

[b] ND: not detected.
